# Organic Compounds Detected in Deciduous Teeth: A Replication Study from Children with Autism in Two Samples

**DOI:** 10.1155/2015/862414

**Published:** 2015-07-29

**Authors:** Raymond F. Palmer, Lynne Heilbrun, David Camann, Alice Yau, Stephen Schultz, Viola Elisco, Beatriz Tapia, Noe Garza, Claudia Miller

**Affiliations:** ^1^Department of Family and Community Medicine, University of Texas Health Science Center, 7703 Floyd Curl Drive, San Antonio, TX 78229-3900, USA; ^2^Department of Analytical and Environmental Chemistry, Southwest Research Institute, 6220 Culebra Road, San Antonio, TX 78238-5166, USA

## Abstract

Biological samples are an important part of investigating toxic exposures and disease outcomes. However, blood, urine, saliva, or hair can only reflect relatively recent exposures. Alternatively, deciduous teeth have served as a biomarker of early developmental exposure to heavy metals, but little has been done to assess organic toxic exposures such as pesticides, plastics, or medications. The purpose of our study was to determine if organic chemicals previously detected in a sample of typically developing children could be detected in teeth from a sample of children with autism. Eighty-three deciduous teeth from children with autism spectrum disorders (ASD) were chosen from our tooth repository. Organic compounds were assessed using liquid chromatography tandem mass spectrometry and gas chromatography methods. Consistent with a prior report from Camann et al., (2013), we have demonstrated that specific semivolatile organic chemicals relevant to autism etiology can be detected in deciduous teeth. This report provides evidence that teeth can be useful biomarkers of early life exposure for use in epidemiologic case-control studies seeking to identify differential unbiased exposures during development between those with and without specific disorders such as autism.

## 1. Introduction

Biological samples are an important part of epidemiological investigations of toxic exposures and their disease outcomes. Blood, urine, saliva, or hair has often been utilized to assess risk factors for a specific disease by comparing the concentrations of some environmental toxic substances between affected and unaffected individuals. While this is a useful approach in some endeavors, these biosamples are only measures of recent exposure and cannot inform about distant past exposures. Therefore, the use of these types of biomarkers is limited.

Alternatively, the use of deciduous teeth has served as a biomarker of early developmental exposure to heavy metals. The mineralization of primary teeth begins prenatally between 14-and 16-week gestation and concludes postnatally at 1.5 to 3 months for incisors, 9 months for canines, and 5.5 to 11 months for molars [1]. It has been demonstrated that metals in circulation, which are present during the period of tooth formation, become incorporated into forming dental tissue and are stored in the mineral component of teeth [2–4].

Deciduous teeth have been used as biomarkers of heavy metal exposure in disease outcome studies for some time. Needleman et al. [5] demonstrated that lead measured in deciduous teeth was associated with lowered cognitive performance in children. Since that landmark study, the concentrations of lead in deciduous teeth have been widely used as a biomarker for lead exposure and body burden in a variety of other studies [6]. Along with lead, other heavy metals such as cadmium, zinc, and copper have also been measured in deciduous teeth [7].

A major development in the assessment of heavy metals using deciduous teeth has been the use of Laser Ablation Electrospray Ionization (LAESI) methods [8]. These methods have been used to determine the timing of exposure during development by taking measurements along the developmental pathway of the tooth. In particular, identification of the perinatal line allows measurements to be assessed which correspond to pre- and postnatal periods. This has been demonstrated with metals such as chromium, iron, mercury, zinc, and antimony [9] and validated with lead, manganese, and other heavy metals [10–13].

Environmental point sources of exposure have been shown to be related to tooth concentrations as a function of age and proximity to the source [14–16]. Further, physiological factors (i.e., gender, tooth type, and weight) as well as behavioral factors (i.e., socioeconomic status, home antiquity, and nail biting habits) have been shown to also explain levels of lead and cadmium in teeth [17, 18].

These studies demonstrate the advantage of analysis of primary teeth for determining early exposures that may be related to specific disease outcomes. Indeed, the conclusions from a recent NATO workshop on the effects of heavy metal pollution on child development recommend that depositions found in teeth can serve as an important tool in relating heavy metal pollution to childhood development outcomes [19]. For example, Adams et al. [20] used deciduous teeth to investigate differential mercury exposure between children with and without autism. Their study demonstrated that there was a higher concentration of mercury in baby teeth of children with autism compared to children without autism. However, a recent study by Abdullah et al. [21] failed to confirm these findings. Other applications of using deciduous teeth as biomarkers of exposure have been with radiation exposure and its relationship to cancer [22].

The majority of studies using deciduous teeth have been on investigating heavy metals and much less has been done to assess* organic* toxic exposures such as pesticides, plastics, or medications. While LAESI methods have been used to precisely determine the period of exposure when assessing metal concentrations, there is concern that heat produced by sectioning the tooth for analysis of semivolatile organic chemicals (SVOC) may vaporize or transform the SVOC on the surface so they will no longer be detectable by LAESI. Notwithstanding, researchers are currently working to overcome the obstacles with LAESI and other imaging methods for use with SVOC. 

*Detection of Organic Chemicals in Teeth*. Gas chromatography/mass spectrometry (GCMS) has been used to determine nicotine in deciduous teeth as a result of household exposure to cigarette smoking [23]. Recently, Camann et al. [24] identified additional semivolatile organic chemicals (SVOC) in deciduous teeth and hypothesized that organic chemicals or their metabolites circulating in the bloodstream during development may absorb into forming dental tissues and remain stored in the tooth thereafter. Chemicals detected by liquid chromatography/tandem mass spectrometry in molars of 21 typically developing children included the endocannabinoid anandamide (86% of children), acetaminophen (43%), and specific metabolites mono-2-ethylhexyl phthalate (MEHP, of plasticizer di-2-ethylhexyl phthalate, 29%), 3,5,6-trichloro-2-pyridinol (TCPy, of organophosphate (OP) insecticide chlorpyrifos, 10%), and 2-isopropyl-6-methyl-4-pyrimidinol (IMPy, of OP insecticide diazinon, 10%). None of these chemicals had previously been detected in teeth.

The use of deciduous teeth with GCMS methods to understand exposures to organic chemicals has great potential for use in epidemiological case-control studies for conditions of unknown etiology such as autism and other childhood disorders. However, unlike LAESI, which can precisely determine the timing of perinatal exposure, GCMS methods are limited to detection of cumulative exposures or at best approximate timing of exposure based on tooth type. Notwithstanding, GCMS methods currently offer a wider range of detectable SVOC that may be hypothesized to be associated with specific health conditions.

The purpose of this study was to replicate the findings of Camann et al. [24] using the deciduous teeth from two different samples of children with autism (in the United States and Mexico). Specifically, we investigated the replication of acetaminophen, arachidonic acid (ARA), 3,5,6-trichloro-2-pyridinol (TCPy, specific chlorpyrifos metabolite), 2-isopropyl-6-methyl-4-pyrimidinol (IMPy, specific diazinon metabolite), diethyl-m-toluamide (DEET), and five monoester phthalate metabolites (monoethyl phthalate (MEP), mono-*n*-butyl phthalate (MnBP), monoisobutyl phthalate (MiBP), monobenzyl phthalate (MBzP), and mono-2-ethylhexyl phthalate (MEHP).

The aforementioned chemicals are pertinent to autism research because several studies indicate that exposure to pesticides [25–27], acetaminophen [28, 29], plastics (Phthalates) [30, 31], and other chemicals [32] is biologically relevant to autism etiology. However, no studies have used objective measures of early life exposure such as deciduous teeth to identify these compounds.

Before future epidemiologic investigations ensue, replication studies are needed to determine whether SVOC concentrations can reliably be detected in diverse samples.

*Post Hoc Analysis*. While this study was not designed to assess validity, mothers who donated their children's teeth also completed an exposure survey which included their reported exposures to acetaminophen, DEET, phthalates, and pesticides. We investigated if the mother's self-reported use or their child's exposure to these compounds would correspond to the concentrations found in their children's teeth.

## 2. Methods

### 2.1. Sample and Recruitment

Seventy-one (71) deciduous teeth, primarily molars and canines (without cavities or fillings), from children with autism spectrum disorders (ASD) were chosen from our tooth repository consisting of 928 children's deciduous teeth. These teeth were obtained through ongoing recruitment efforts as part of our IRB-approved pilot studies on autism through the University of Texas Health Science Center (UTHSCSA). A component of our program involves an established biological tissue biorepository from families of children with and without autism. We have recently added the collection of deciduous teeth through collaborative efforts with the Interactive Autism Network (IAN) [33]. To control potential bias from using a mixed sample, we chose children recruited from the Interactive Autism Network (IAN) whose diagnoses of autism have been verified [33, 34].

As a cross-cultural comparison in a separate analysis, we also included 12 teeth from autistic children obtained through UTHSCSA collaborations with an autism parent group from Matamoros, Mexico.

### 2.2. Laboratory Methods

83 tooth crowns were pulverized, extracted in batches of approximately 20 samples, and analyzed for acetaminophen, ARA, DEET, TCPy, IMPy, and MEHP using modified methods from Camann et al. [24]. A dentist identified each deciduous tooth crown. The pulp and any fillings, attached roots, and/or cavities were removed with a scalpel, engraving tool, and/or heatless wheel. The tooth was gently swirled in dichloromethane (DCM), with the wash retained as a quality measure to evaluate external tooth contamination. Each prepared tooth crown (enamel + dentin) was pulverized with a mortar and pestle to a fine powder and weighed.

Prior to extraction, a 50 mg pulverized tooth aliquot was spiked with acetaminophen-*d*_4_ and MEHP-^13^C_4_ and conditioned for 24 hours. The tooth aliquot was separately extracted under neutral and acidic conditions to enhance the extraction efficiency of the target analytes. The first extraction occurred through sonication with 0.5 mL acetonitrile. After removing the acetonitrile fraction, the tooth powder was acidified with glacial acetic acid and equilibrated overnight, with a second sonication extraction conducted using acetonitrile. Both acetonitrile extracts were concentrated to 50 *μ*L prior to analysis. Electrospray ionization liquid chromatography tandem mass spectrometry (LC/MS/MS) in multiple-reaction monitoring mode was used to determine the concentrations of most targeted chemicals in the pulverized tooth samples. Acetaminophen and DEET were measured in positive mode, and TCPy, IMPy, and the phthalate metabolites in negative mode. A matrix blank with 50 mg of pulverized kiln-fired synthetic hydroxyapatite was extracted with each batch of tooth samples and analyzed to assess laboratory introduced contamination. A matrix spike of all target analytes, into a second 50 mg aliquot of two pulverized teeth, was extracted and analyzed to assess measurement accuracy (i.e., analyte extraction efficiency) in these teeth. The organic wash portion was analyzed as a QC measure to assess external contamination for specific samples containing high levels of detected target analytes.

To determine the concentration of ARA and other fatty acids (IAN sample only), a second 50 mg aliquot of each pulverized tooth was spiked with triheneicosanoin, decalcified with EDTA, and extracted three times with chloroform. Tridecanoic and tricosanoic acids were added as internal standards, and the extract was blown down to dryness, saponified, and methylated. The fatty acid methyl esters were extracted with saturated sodium chloride and isooctane, and the concentrated isooctane extract was analyzed for ARA by gas chromatography mass spectrometry in selected ion monitoring mode.

### 2.3. Mothers Reported Use and Exposure to Acetaminophen

Seven dichotomous items on the survey, indicating use (=1) or no use (=0) of acetaminophen, were summed to create a total exposure score. The items captured assessed (1) mother's use during pregnancy, (2) child's use from 0 to 6 months, (3) child's use from 7 to 12 months, (4) child's use from 13 to 18 months, and (5) around the time before or after Measles Mumps and Rubella (MMMR) vaccination (12–15 months). We also utilized information about the effectiveness of acetaminophen based on the mother's report of whether it works for her or her child (1 = works well versus 0 = works a little or not at all), reasoning that if this analgesic worked, then it would be used. Scores ranged from 0 to 7. A dichotomous variable was then formed using a median split roughly corresponding to a 50% split of the distribution (0 = lower half, *n* = 28 versus 1 = upper half, *n* = 43). This self-report exposure variable was submitted to a chi-square analysis, using Fisher's exact test, to determine if the two self-reported exposure groups had statistically different rates of acetaminophen detection in teeth.

### 2.4. Mother Reported Use and Exposure to Insect Repellent (DEET)

This was constructed from five items asking the number of times insect repellant was used on their child at less than 1 years old, 1-2 years old, 2-3 years old, 3-4 years old, and 4-5 years old. Each response was scored 0 for no use during that time, 1 = 1–4 times, 2 = 5–9 times, and 3 = more than 10 times. Since 83 percent said they had used repellants sometime during their child's life, a sum score was used to reflect the frequency of repellant use summed over the five time frame items. This variable was also dichotomized based on a median split.

These self-reported insect repellant exposure variables were submitted to a chi-square analysis using Fisher's exact test to determine if the highest self-reported exposure group category had statistically different rates of DEET detection in teeth compared to the lowest reported exposure. We also used a Tobit regression (suitable for data that are left truncated due to laboratory detection limits) to assess the linear association between the continuous self-reported score and the continuous DEET tooth concentration, adjusted for parental age and gender.

### 2.5. Mother Reported Use and Exposure to Pesticides

This was constructed from responses to three areas of usage. (1) Did anyone use sprays, dusts, powders, mothballs, or foggers (including a pest control service) in your home or place of work to kill bugs? (2) Did anyone use a lawn service or apply bug or weed killers on your yard, plants, or trees? (3) Did anyone use sprays, dusts, powders, soaps/shampoos, or skin applications for fleas or ticks on your pets? Each of the three questions had response options of 0 = not at all, 1 = once or twice, 2 = three to five times, and 3 = six or more times covering three developmental time periods: before pregnancy, during pregnancy, and from birth to six years old. A dichotomous summary variable based on a median split was formed to reflect high and low self-reported exposure. Chi-square analysis was performed with this dichotomous self-reported use and a dichotomous variable reflecting pesticide metabolites detection in teeth.

### 2.6. Mothers Reported Use and Exposure to Phthalates

Cumulative self-reported exposure to phthalates was determined using questions asking whether or not the child was exposed to fumes/chemicals from* new paint, new floors/cabinets (stains *and* paint strippers), new carpet, new walls/drywall, or carpet cleaning* during three developmental periods: three months before pregnancy, during pregnancy, and during the child's life. Scores were summed to produce a total self-reported exposure score. A dichotomous variable was formed based on a median split and used as a predictor in a Tobit regression model with phthalates as an outcome.

## 3. Results

### 3.1. IAN Sample

Table 1 describes the sample used for this study. The teeth from IAN children with autism are of parents who are largely middle class non-Hispanic white families. The Matamoros samples of children with autism are Hispanic families with comparably lower income and education.

Table 2 shows the distribution of chemicals we sought to measure. There was a 44% detection rate for acetaminophen (31/71) and a 75% detection rate for DEET (53/71). The detection rate for both of the organophosphate insecticide metabolites TCPy and IMPy was 13%. Detection rates of the low molecular weight monoester phthalate metabolites were (monoethyl phthalate (MEP) (100%), mono-*n*-butyl phthalate (MnBP) (86%), and monoisobutyl phthalate (MiBP) (70%). Relatively smaller detection rates were found for monobenzyl phthalate (MBzP) (6%) and mono-2-ethylhexyl phthalate (MEHP) (36%). The detection rates of linoleic acid (LA) and arachidonic acid (ARA) were 100 and 85 percent, respectively. For alpha-linolenic acid (ALA) and docosahexaenoic acid (DHA), detection rates were much lower at 21 and 20 percent, respectively.

### 3.2. Matamoros Sample

Table 3 shows the distribution of chemicals from Matamoros. There was a 42% detection rate for acetaminophen (5/12) and a 100% detection rate for DEET. The detection rate for TCPy was zero and for IMPy was 17% (2/12).

Detection rates of the low molecular weight monoester phthalate metabolites were (monoethyl phthalate (MEP) (100%), mono-*n*-butyl phthalate (MnBP) (66%), and monoisobutyl phthalate (MiBP) (100%). Detection rates were found for monobenzyl phthalate (MBzP) (0%) and mono-2-ethylhexyl phthalate (MEHP) (100%).

### 3.3. Post Hoc Analysis: Mother Reported Use and Exposure to Acetaminophen

Among those self-reporting the highest cumulative exposures, 53.5 percent (*n* = 23/43) had detectable amounts of acetaminophen in teeth compared to 28.6 percent (*n* = 8/28) in the lower self-reported exposure group (Fisher exact test = *p* < .041). In a logistic regression model adjusting for age, gender, and tooth type, those reporting higher exposures were 3.18 times more likely to have a detection of acetaminophen than those with lower self-reported exposures (*p* < .03).

### 3.4. Mother Reported Use and Exposure to Insect Repellant (DEET)

The distribution of DEET in teeth was highly skewed. A square root transformation greatly improved the distribution suitable for parametric tests. We also created a dichotomous variable where 1 = any detection and 0 = no detection of DEET.

Chi-square analysis showed that 90.9 percent of those in the higher self-reported use category had detectable amounts of DEET in teeth compared to 63.3 percent detection in the lower self-reported use group. This difference was statistically significant (Fisher exact test, *p* < .01). DEET tooth concentration was regressed on self-reported insect repellant use (on the children only) in three models: (a) self-reported use before 2 years old, (b) over 2 years old, and (c) across the entire developmental timespan. After adjustment for parental age, child age and gender, and tooth type, exposure under 2 years old yielded a .34 standardized regression coefficient (*p* < .01) (*r*-square = .18). Exposures after three years old yielded a .38 standardized regression coefficient (*p* < .001) (*r*-square = .21). Exposures during the entire developmental period yielded a .36 standardized regression coefficient (*p* < .005) (*r*-square = .20). Tobit regression reveals that the highest self-reported tertile level of exposure had 55.7 ng/g higher levels of DEET than the lowest one third (*p* = .01).

### 3.5. Mother Reported Use and Exposure to Pesticides

The distribution of the organophosphate insecticide metabolites TCPy and IMPy in 71 teeth is shown in Table 2. The detection rate of both metabolites was 13% in the sample (9/71), with no detection in 62 of the teeth. Both variables were dichotomized to form a binary variable 0 = no detection (*n* = 62) and 1 = detection (*n* = 9). We also summed both insecticide metabolites to form another dichotomous variable, where 0 = no detection (*n* = 55) and 1 = detection (*n* = 16). There was no significant association between any of the self-reported pesticide usage variables and any of the chlorpyrifos and diazinon metabolite tooth measures.

### 3.6. Mother Reported Use and Exposure to Phthalates

There was no significant association between self-reported fumes/chemicals exposure from listed products and measured phthalate metabolites in teeth for MnBP or MEP. There was, however, a significant association for MiBP. The Tobit regression results indicated that relative to the low self-reported group, the high self-reported group had 94 ng/g higher concentration of MiBP in teeth. When combining all phthalates together, the Tobit regression results indicated that relative to the low self-reported group the high self-reported group had 97 ng/g higher concentration of MiBP in teeth; however this was marginally significant (*p* < .08).

## 4. Discussion

Consistent with the prior report from Camann et al. [24], which used 21 deciduous teeth from typically developed children, we have demonstrated that specific SVOC can be detected in deciduous teeth from children with autism in two different populations. Despite demographic differences in the two samples, there were similar rates of detection for all chemicals with slightly higher detection rates of phthalates in the Matamoros sample.

This provides evidence that teeth are useful biomarkers of exposure to these SVOC compounds and therefore can be used in properly matched case-control studies to investigate potential differences in exposure between cases (e.g., children with autism) and controls (e.g., children without autism).

This method of assessing early life environmental exposures is relevant to a wide range of diseases suspected to involve environmental triggers. Prenatal exposures to polycyclic aromatic hydrocarbons, pesticides, secondhand smoke, diester phthalates, and polybrominated diphenyl ethers are linked to reduced fetal growth and developmental problems in young children [35–39]. Similarly, previous studies report a link between* in utero* organochlorine pesticide exposure and impaired neurodevelopment in childhood [40], and there is emerging evidence of neurobehavioral consequences for infants and children who have been exposed to even low levels pesticides [39, 41, 42]. Taken together, these studies suggest that there are multiple chemicals that may be associated with increased risks for ASD [43].

Chlorpyrifos has been shown to disrupt development of the serotonin neurotransmitter system; this is similar to serotonin system dysfunction which has been implicated in autism etiology [44–46]. Chlorpyrifos is widely used in agriculture and remains an exposure of concern for children through diet even though the EPA has restricted its indoor use. There are additional reports addressing established associations between ubiquitous environmental chemicals, including pesticides [47] with effects on neurodevelopment and/or immune system regulation [48]. For example, an elevated risk for ASD has been attributed to the commonly used pesticide permethrin, based on self-reports of exposure [49].

Given the male predominance in ASD, it is also relevant that phthalates are potential endocrine disruptors which can interfere with normal male development [50]. Diethylhexyl phthalate (DEHP), one of the most common phthalates present indoors, is often used as a plasticizer in polyvinylchloride (PVC) materials, such as PVC flooring and water pipes. Two recent small-scale studies have linked DEHP with autism [30, 51].

Prenatal acetaminophen exposure has been associated with asthma symptoms at age five [52], while acetaminophen use within the first 18 months after birth may be associated with increased susceptibility to autism [53, 54]. Acetaminophen is an indirect agonist of the endocannabinoid system, affecting levels of the endocannabinoids anandamide and 2-arachidonoylglycerol. Anandamide, an endogenous activator of the endocannabinoid system, and 4-aminophenol, the active metabolite of acetaminophen, have similar toxic effects on developing cortical neurons [55].

The mode of action, metabolic activation, and detoxification of many ubiquitous environmental pesticides are well known and have been linked to the disruption of endocrine activity and to disruption in the function of the primarily inhibitory neurotransmitter, gamma-aminobutyric acid (GABA) [56, 57]. Various studies demonstrate abnormalities of the glutamate and GABA systems in the brain and serum of subjects with autism [58, 59]. For example, there is a significant decrease in GABA and various GABA subunit receptor-binding sites in brain tissues of subjects with autism when compared with controls [60]. Similarly, perinatal exposure to these environmental pesticide compounds appears to be associated with ASD through the disruption of GABA function; there are similar reports that pesticides interfere with development by disrupting thyroid function [25, 31].

In sum, there is a substantial amount of converging evidence to suggest that environmental chemicals may play a role in ASD risk and/or etiology by acting independently or through interactions with genetic vulnerabilities.

The post hoc analysis results indicate that mother's self-report of her child's usage/exposure to acetaminophen and products containing DEET and phthalates are consistent with detection and concentration levels in the child's deciduous tooth. While compelling, this provides limited support for concurrent validity. Until additional studies are completed, we can make no claim about the developmental timing of exposure to the aforementioned chemicals found in deciduous teeth using the laboratory methods described in this paper. It would therefore be useful for future validation studies to be performed involving laboratory experimental models that manipulate* in vivo* timing of exposures. While LAESI methods have been used to precisely determine the period of exposure when assessing metal concentrations, there is concern that heat produced by the necessary sectioning of the tooth may vaporize or transform the SVOC on the surface so they will no longer be detectable. However, “soft ionization” imaging techniques such as Matrix Assisted Laser Desorption Ionization Time-of-Flight (MALDI-TOF) mass spectrometry may circumvent this issue. Researchers are currently working to overcome the obstacles with sectioning methods for use with SVOC.

There are thousands of potential environmental chemicals in circulation to which humans are exposed [61]. Even within certain chemical classes, there are hundreds of structurally related chemicals that have potential relevance to ASD. Therefore, exploratory and targeted GC and LC/MS studies with pulverized teeth to identify candidate chemicals will be an important first step. Chemicals identified in these initial studies can then be targeted in future imaging studies to fine-tune the time period of exposure. We feel these initial investigations are important for informing future epidemiologic research that can more precisely identify the timing of exposures between cases and controls.

*Limitations*. While we have demonstrated that chemicals relevant to ASD can be detected in deciduous teeth and are associated with mothers' self-reported exposures, our results are limited in generalizability—largely due to the sample consisting entirely of children with ASD. Future studies that include more diverse participants and neurotypical children as controls will allow case/control comparisons.

Further, until additional studies are completed, we make no claim about the developmental timing of exposure (pre- or postnatal) to the aforementioned chemicals found in deciduous teeth using the laboratory methods described in this paper. Studies incorporating both unbiased environmental exposure measures (e.g., deciduous teeth) and measures of individual variation in the ability to biologically detoxify specific toxins (e.g., gene expression assessments) would be the next step in understanding gene-environment interactions that could effectively inform various prevention efforts.

## Figures and Tables

**Table 1:** Descriptive statistics of the study sample of children with ASD.

	Mean (SD) or % IAN *n* = 71	Mean (SD) or % Matamoros *n* = 12
Parent characteristics		
Mother's age^1^ in years	43.6 (6.0)	38.3 (8.9)
Father's age^*∗*^ in years	45.7 (6.0)	41.9 (10.3)
Mothers born in USA	86.4%	0%^*∗∗∗*^
Fathers born in USA	84.6%	9%^*∗∗∗*^
Married	79.1%	75%
Four-year college degree or higher	58.1%	25%^*∗∗*^
Household yearly income equal to or greater than $50,000	73.4%	0%^*∗∗∗*^
Child characteristics		
Age^1^ (years)	11.5 (4.4)	11.1 (3.9)
Non-Hispanic White	82.1%	0%^*∗∗∗*^
Male	71.1%	92%^*∗*^

^1^Age at tooth donation.

^*∗*^ *p* < .05, ^*∗∗*^ *p* < .01, ^*∗∗∗*^ *p* < .001.

**Table 2:** Detection frequency and concentration distributions of SVOC in a deciduous tooth crown of 71 IAN children with ASD.

Analyte (parent)	Mean (ng/g)	Number of teeth	Detections		Concentration (ng/g)					
			Number	Percent	P_25_	P_50_	P_75_	P_90_	P_95_	Max
LC/MS/MS-detected analytes										
Acetaminophen	0.98	71	31	44%	<1.4	<1.4	5.1	24.4	52.5	1050
DEET	82.2	71	53	75%	<4.3	13.7	47.5	101	198	3270
TCPy (chlorpyrifos)	0.88	71	9	13%	<1.3	<1.3	<1.3	1.9	8.7	18.5
IMPy (diazinon)	0.96	71	9	13%	<1.0	<1.0	<1.0	0.4	2.8	56.7
MEP^*∗*^ (DEP)	268.6	31	31	100%	56.5	138	235	870	1290	1720
MnBP (DnBP, BzBP)	229.0	71	61	86%	11.0	34.0	157	730	1270	3250
MiBP (DiBP)	70.24	71	50	70%	<6.0	19.7	62.7	186	342	790
MBzP (BzBP)	2.39	71	4	6%	<10.0	<10.0	<10.0	<10.0	25.4	74.3
MEHP (DEHP)	47.0	56	20	36%	<10	<10	32.9	214	319	715

Polyunsaturated fatty acids	Mean *μ*g/g	Number of teeth	Detections		Concentration (*μ*g/g)					

LA, C18:2 n6	14.1	71	71	100%	4.42	7.58	17.0	34.3	61.2	92.1
ALA, C18:3 n3	0.70	71	15	21%	<1.0	<1.0	<1.0	1.96	3.60	15.4
ARA, C20:4 n6	2.60	71	60	85%	1.29	1.82	3.49	5.23	8.66	15.0
DHA, C22:6 n3	0.31	71	14	20%	<1.0	<1.0	<1.0	1.52	2.09	2.41

^*∗*^MEP reported after matrix blank subtraction.

Recovery range of equilibrated spiked surrogates from the 71 teeth was 8–92% for acetaminophen-*d*_4_, 55–222% for MEHP-^13^C_4_, and 30–163% for C21:0.

**Table 3:** Detection frequency and average concentrations of sVOC in deciduous tooth crowns of 12 children with ASD from Matamoros.

Analyte (parent)	Mean (ng/g)	Number of teeth	Detections		Concentration (ng/g)					
			Number	Percent	P_25_	P_50_	P_75_	P_90_	P_95_	Max
LC/MS/MS-detected analytes										
Acetaminophen	1.69	12	5	42%	<0.4	<0.4	0.89	1.04	4.85	4.85
DEET	39.3	12	12	100%	10.8	16.7	32.3	58.5	242.5	242.5
TCPy (chlorpyrifos)	0	12	0	0	<1.3	<1.3	<1.3	<1.3	<1.3	<1.3
IMPy (diazinon)	2.02^*∗*^	12	2	17%	<1.0	<1.0	<1.0	1.85	2.18	2.18
MEP^*∗*^ (DEP)	115.0	12	12	100%	75.9	115.1	151.1	176.5	207.5	207.5
MnBP (DnBP, BzBP)^*∗*^	22.92	12	8	66%	9.1	9.7	29.9	42.2	50.4	50.4
MiBP (DiBP)	55.25	12	12	100%	11.43	41.9	85.3	135.8	144.6	144.6
MBzP (BzBP)	0	12	0	0	<10.0	<10.0	<10.0	<10.0	<10.0	<10.0
MEHP (DEHP)	198.5^*∗*^	12	2	100%	22.5	67.6	148.5	383.8	1347.4	1347.4

^*∗*^Significantly different (*p* < .01) than non-Hispanic White sample in Table 2.
